# Supplementary material for: Quantification of critical particle distance for mitigating catalyst sintering
Source: Nat Commun. 2021 Aug 11;12:4865. doi: 10.1038/s41467-021-25116-2 (PMC8358017; doi:10.1038/s41467-021-25116-2)
Supplement: Supplementary file 1 — Supplementary Information [file 41467_2021_25116_MOESM1_ESM.pdf]

Supplementary Information for

Quantification of critical particle distance for mitigating catalyst sintering

Peng Yin[1,2]†, Sulei Hu[1,3]†, Kun Qian[1,3], Zeyue Wei[1,3], Le-Le Zhang[1,2], Yue Lin[1]*, Weixin Huang[1,3], Haifeng Xiong[4], Wei-Xue Li[1,3]*, Hai-Wei Liang[1,2]*

[1]Hefei National Laboratory for Physical Sciences at the Microscale, University of Science and Technology of China, Hefei 230026, China

[2]Department of Chemistry, University of Science and Technology of China, Hefei 230026, China

[3]Key Laboratory of Surface and Interface Chemistry and Energy Catalysis of Anhui Higher Education Institutes, Department of Chemical Physics, University of Science and Technology of China, Hefei 230026, China

[4]State Key Laboratory of Physical Chemistry of Solid Surfaces, College of Chemistry and Chemical Engineering, Xiamen University, 361005 Xiamen, China

†These authors contributed equally to this work.

*email: linyue@ustc.edu.cn; wxli70@ustc.edu.cn; hwliang@ustc.edu.cn

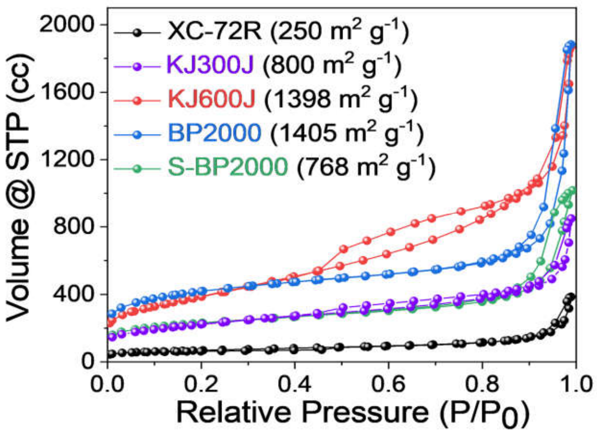

Supplementary Figure 1. N_2 adsorption/desorption of the five carbon black supports.

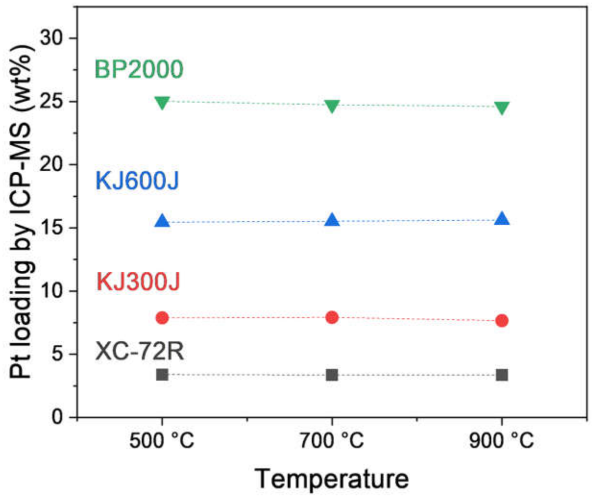

Supplementary Figure 2. Pt loading of the four carbon black supported catalysts upon thermal treatments at 500, 700, and 900 °C.

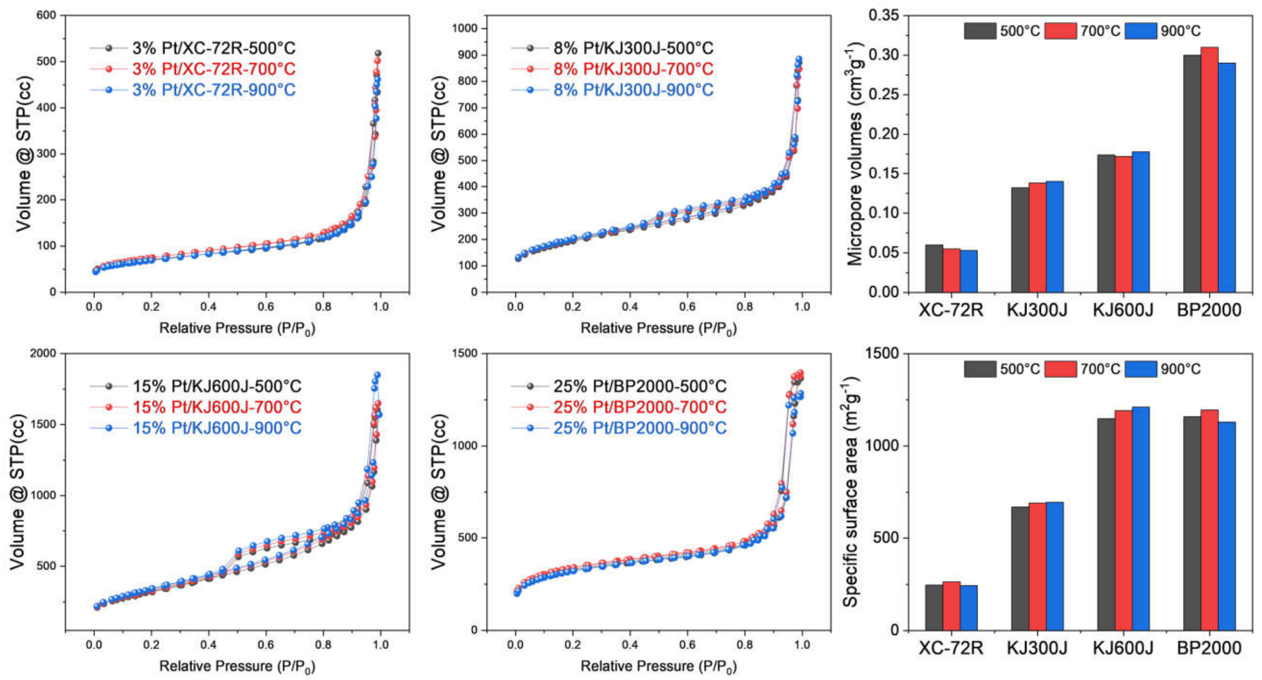

Supplementary Figure 3. Isothermal physisorption curves, the corresponding micropore volumes and specific surface area of the carbon black supported catalysts upon thermal treatments at 500, 700, and 900 °C.

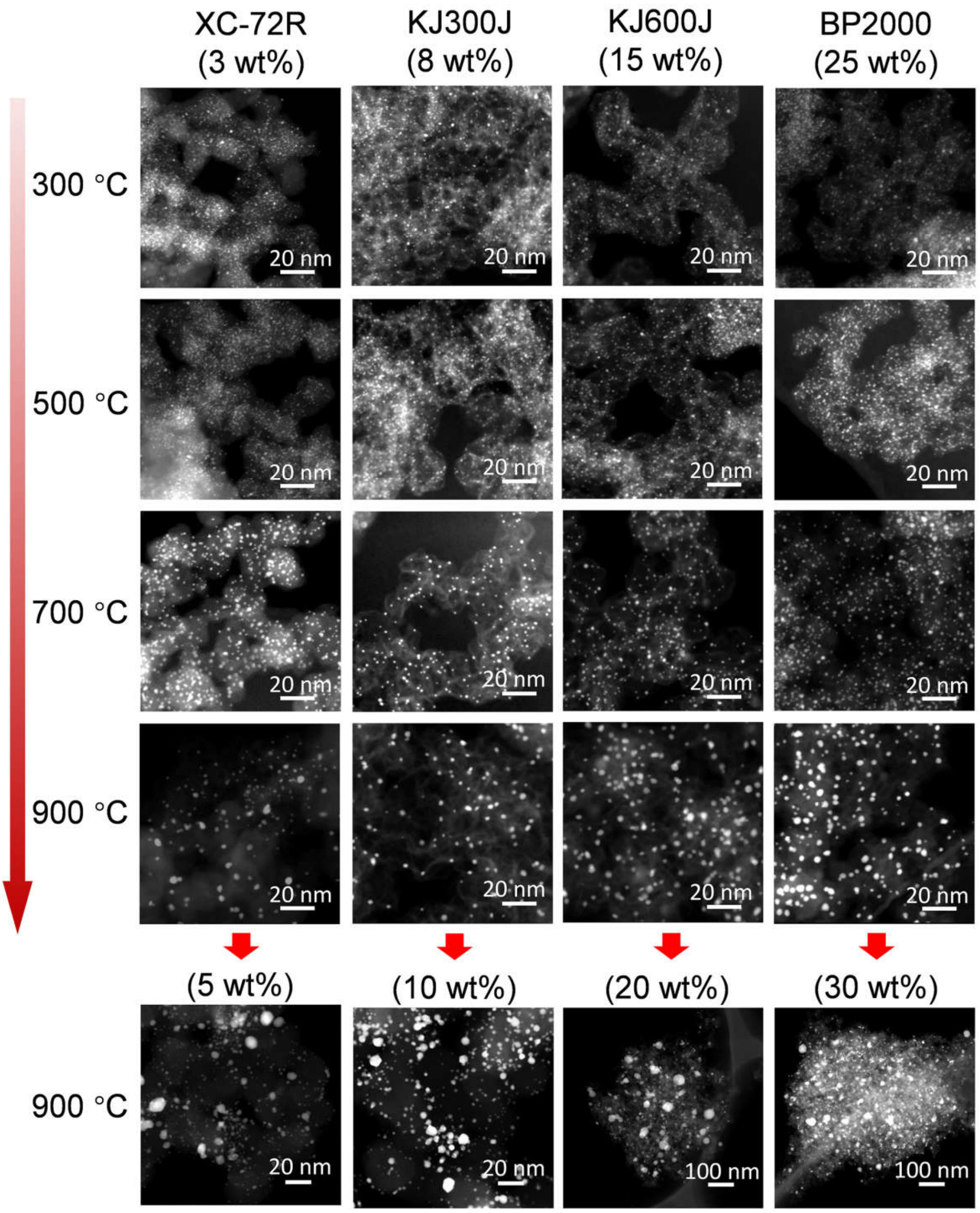

Supplementary Figure 4. HAADF-STEM images of the Pt/C catalysts after sintering tests at different temperatures of 300, 500, 700, and 900 °C in 5% H_2/Ar for 120 min. Abnormally large particles were observed once the Pt loading increased by 2~5% on the basis of the corresponding upper limit loading for each support.

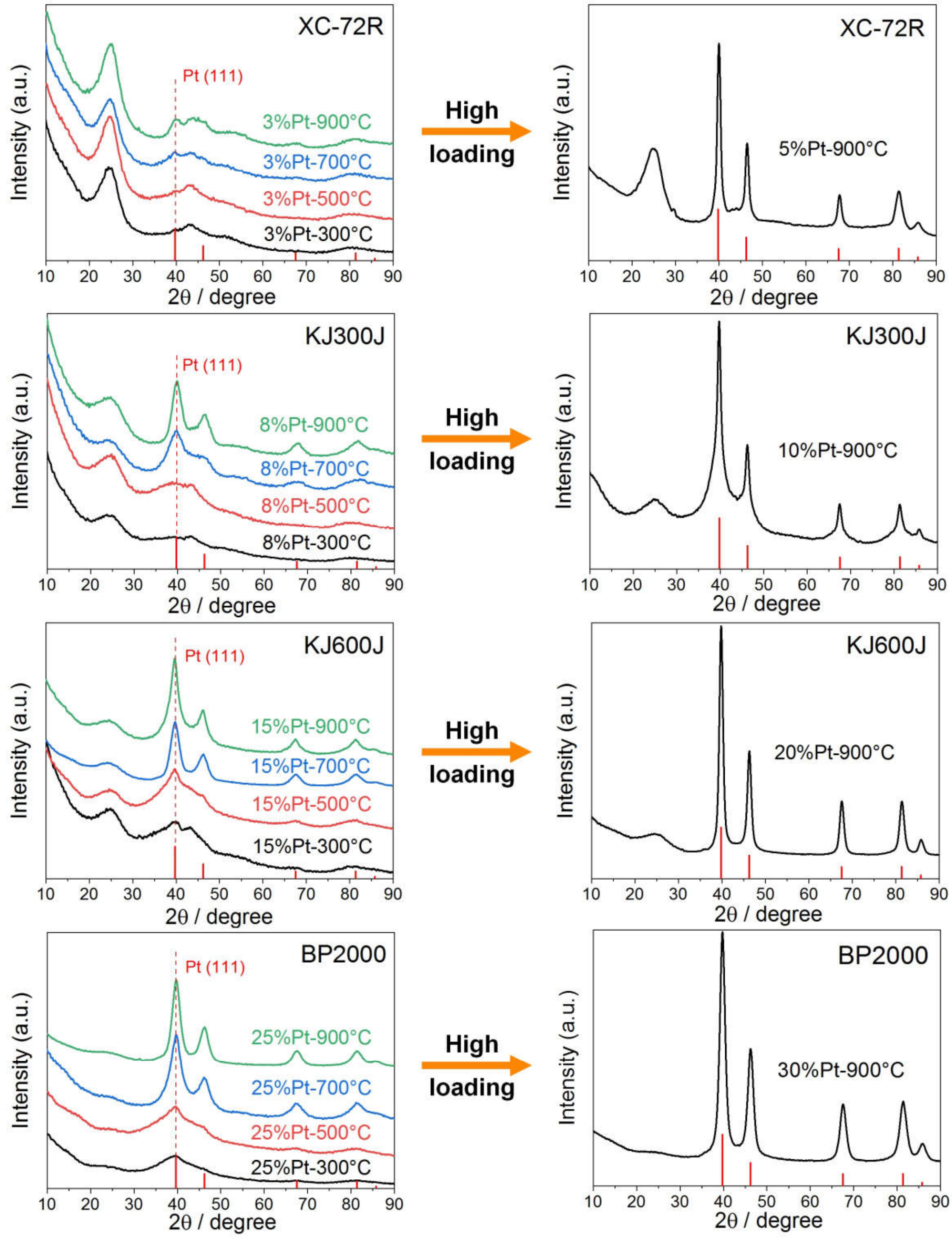

Supplementary Figure 5. XRD patterns of the Pt/C catalysts after sintering tests at different temperatures of 300, 500, 700, and 900 °C in 5% H_2/Ar for 2 h, showing the loading-dependent sintering behavior. The standard peaks of Pt are shown in red lines.

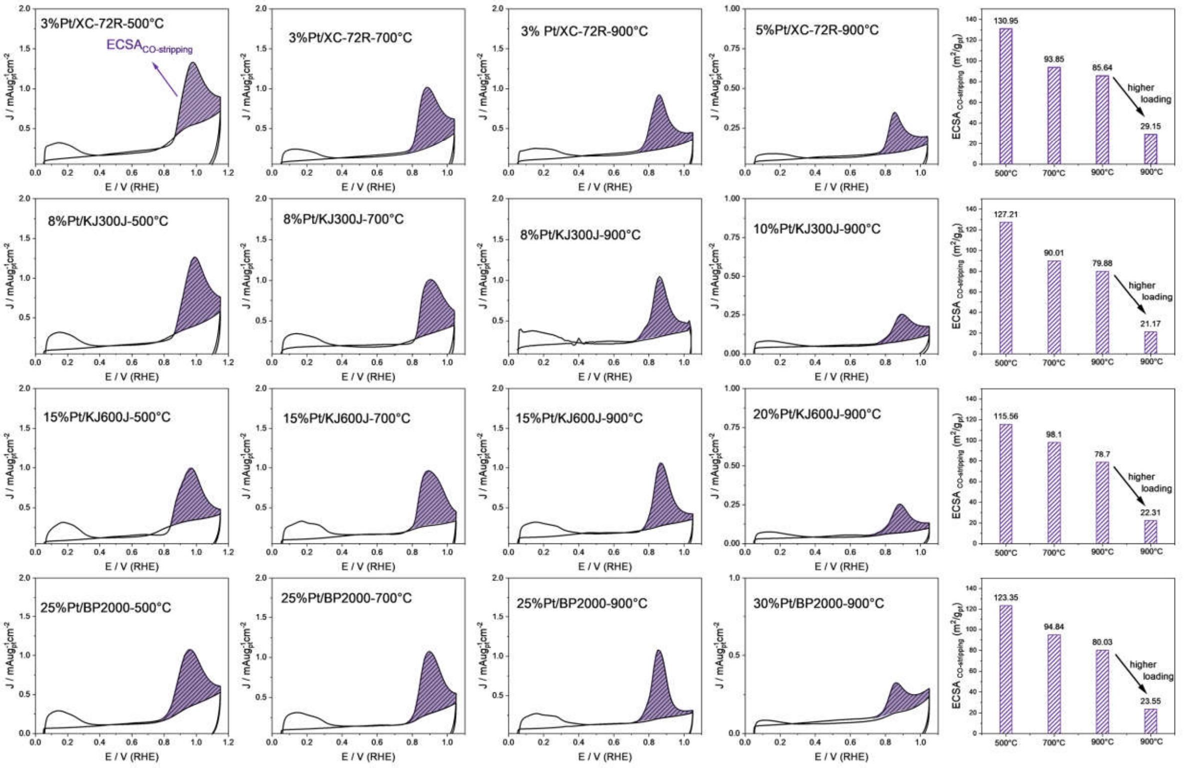

Supplementary Figure 6. CO-stripping of different Pt/C catalysts and the corresponding ECSA.

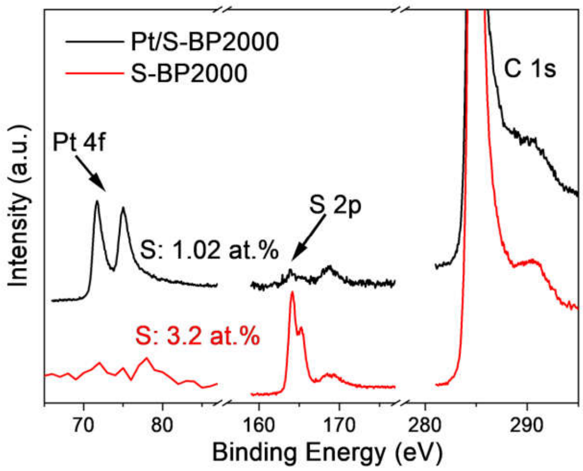

Supplementary Figure 7. XPS survey spectra of the S-BP2000 support and the Pt/S-BP2000 catalyst after annealing treatment at 900 °C.

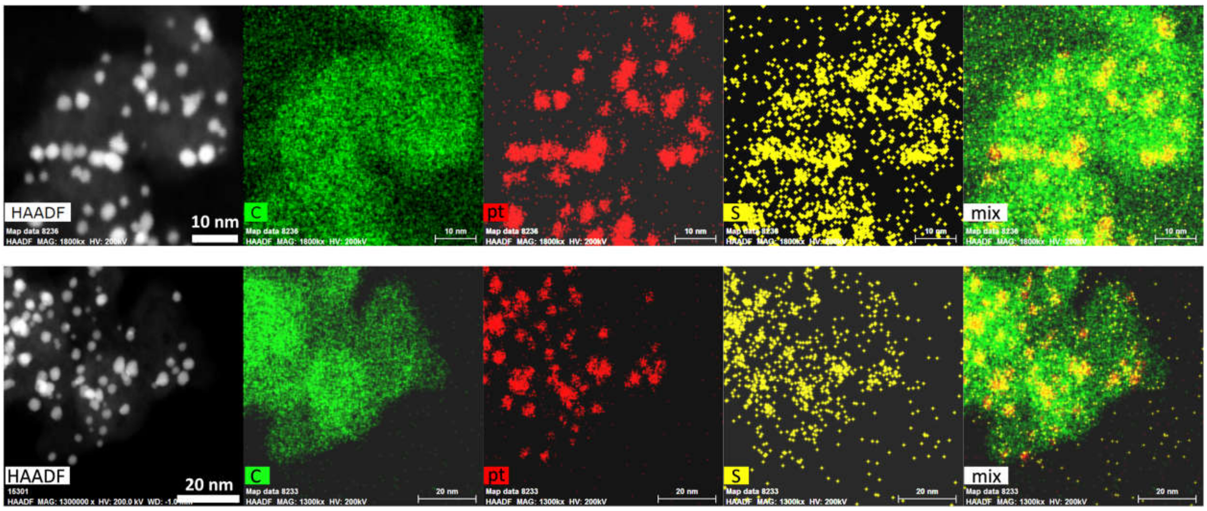

Supplementary Figure 8. EDS mapping of Pt/S-BP2000 after the treatment at 900 °C, showing the spatial overlapping of Pt and S elements.

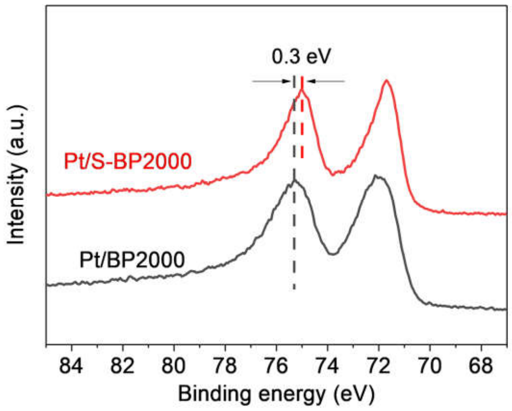

Supplementary Figure 9. XPS spectra of Pt 4f on Pt/S-BP2000 and Pt/BP2000.

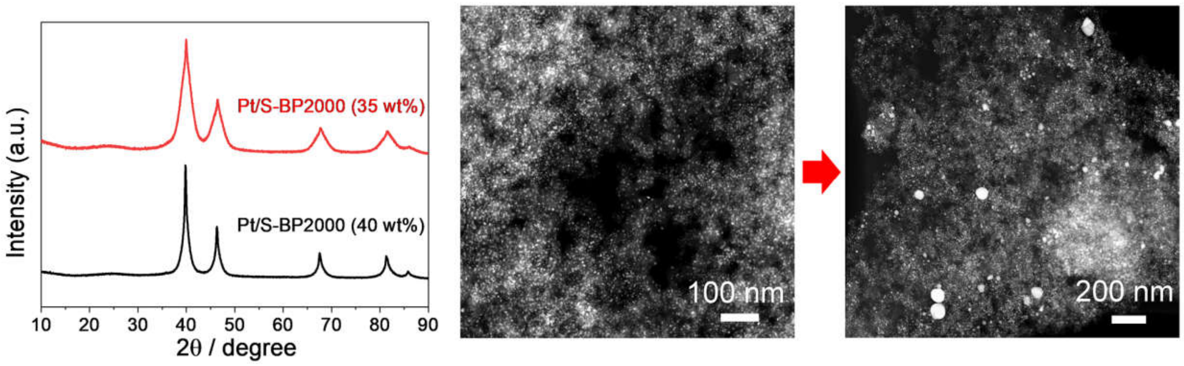

Supplementary Figure 10. XRD patterns and HAADF-STEM images of 35%Pt/S-BP2000 and 40%Pt/S-BP2000 after sintering tests at 900 °C in 5% H_2/Ar for 2 h, showing the improvement of upper limit loading by strengthening the metal-support interaction by sulfur doping on carbon supports.

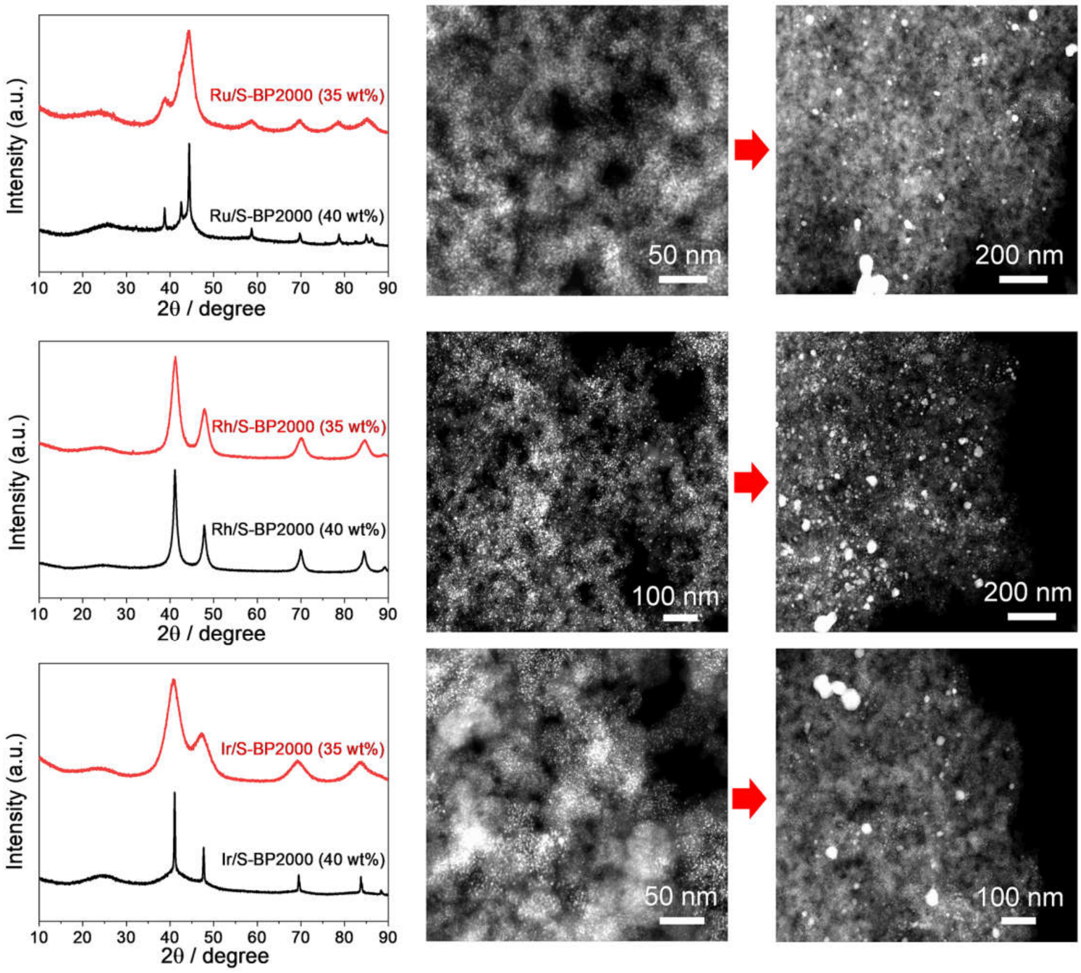

Supplementary Figure 11. XRD patterns and HAADF-STEM images of S-BP2000 supported Ru, Rh, Ir catalysts after sintering tests at 900 °C in 5% H_2/Ar for 2 h, showing the universality of the concept of controlling particles distance for mitigating catalyst sintering.

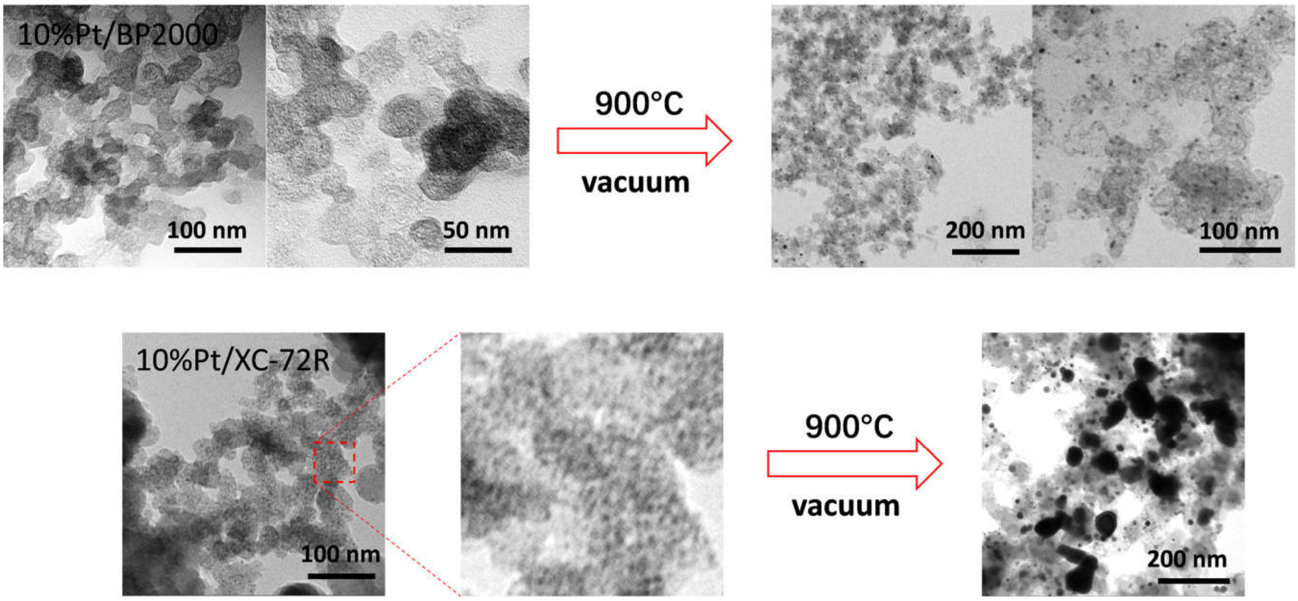

Supplementary Figure 12. The sintering test of Pt/BP2000 and Pt/XC-72R in tube furnace under vacuum condition, showing similar particle distance behavior in 5% H_2/Ar.

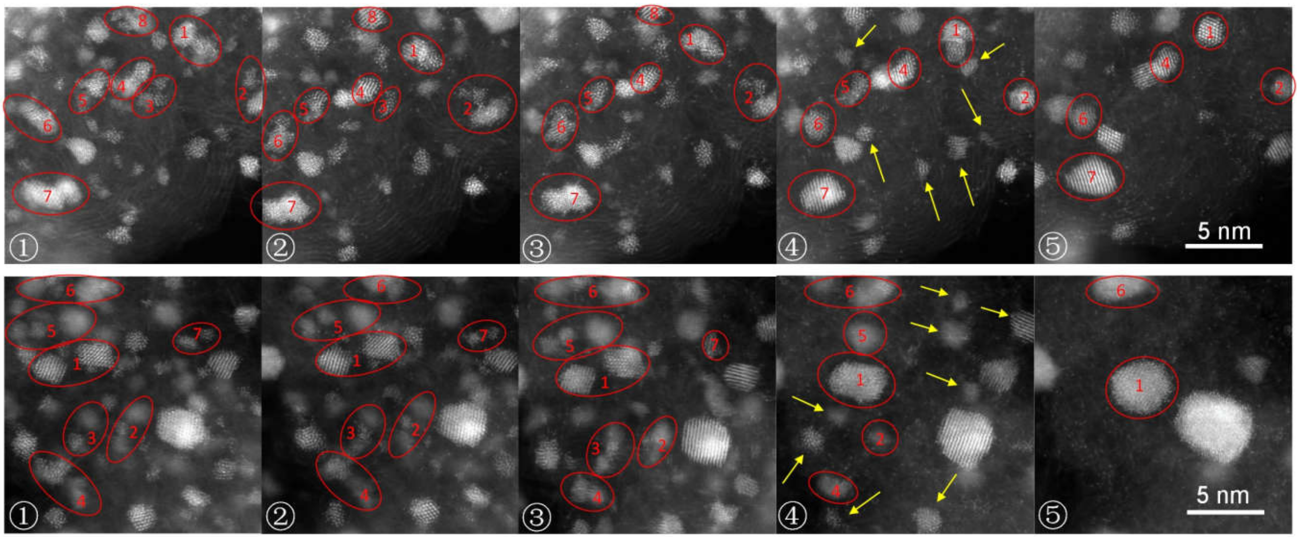

Supplementary Figure 13. In situ aberration-corrected HAADF-STEM images of 10%Pt/XC-72R ($d < d_c$) at five sintering stages shown in Fig. 4c. The particle marked with red circles were tracked and counted for OR or PMC path. The yellow arrows indicate the particles disappeared in the next stage.

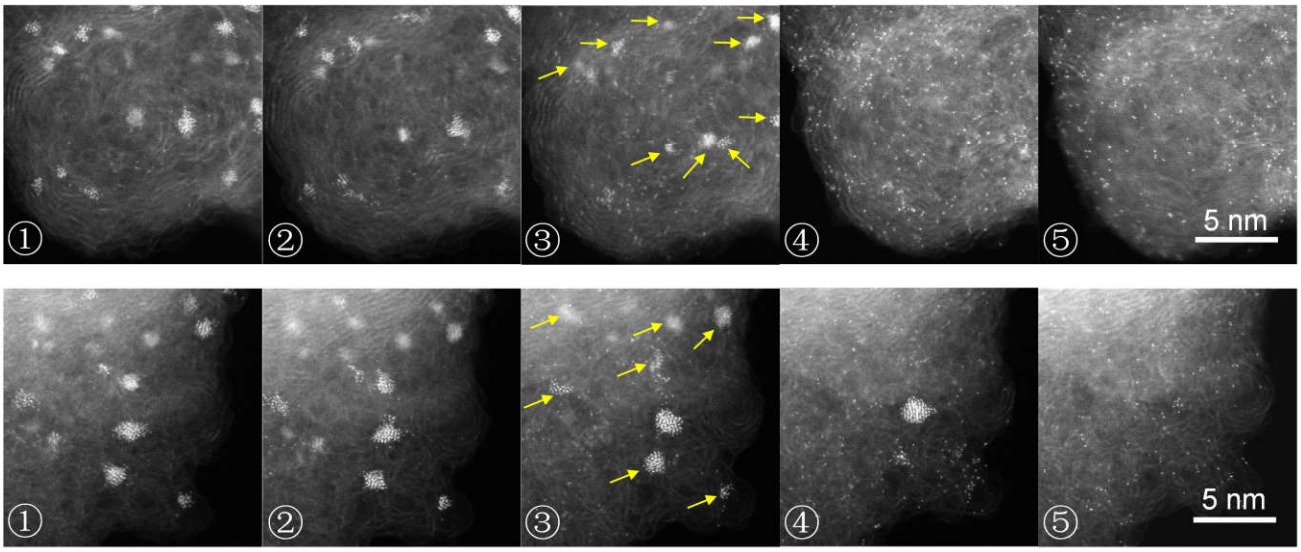

Supplementary Figure 14. In situ aberration-corrected HAADF-STEM images of 1%Pt/XC-72R ($d > d_c$) at five sintering stages shown in Fig. 4c. The yellow arrows indicate the particles disappeared in the next stage.

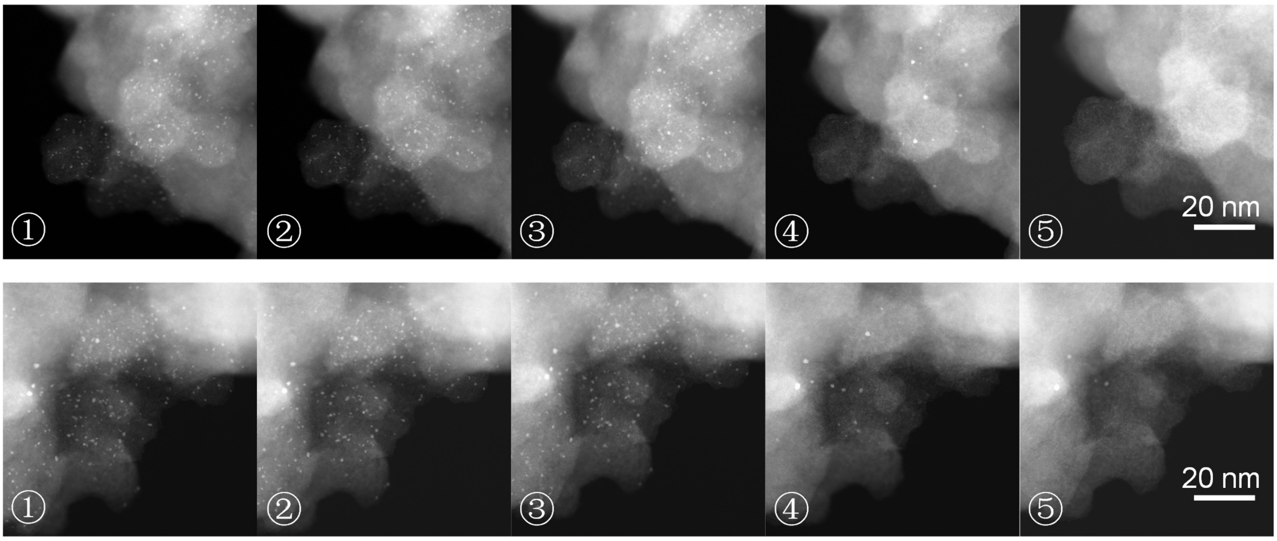

Supplementary Figure 15. In situ aberration-corrected HAADF-STEM images of 1%Pt/XC-72R ($d > d_c$) in large region at five sintering stages shown in Fig. 4c.

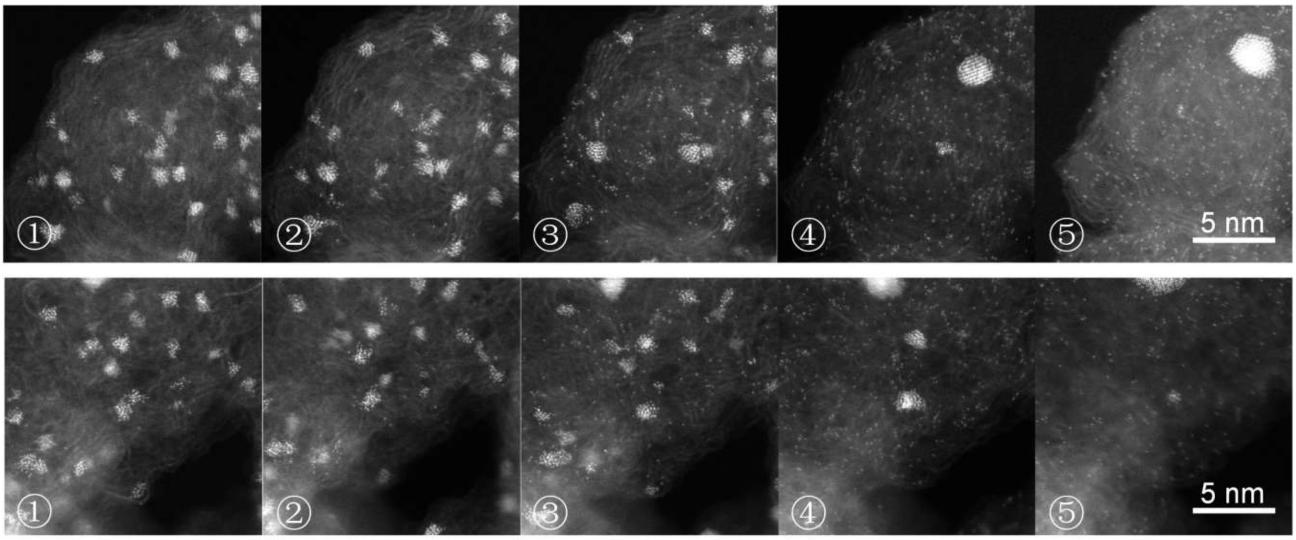

Supplementary Figure 16. In situ aberration-corrected HAADF-STEM images of 10%Pt/BP2000 ($d > d_c$) at five sintering stages shown in Fig. 4c.

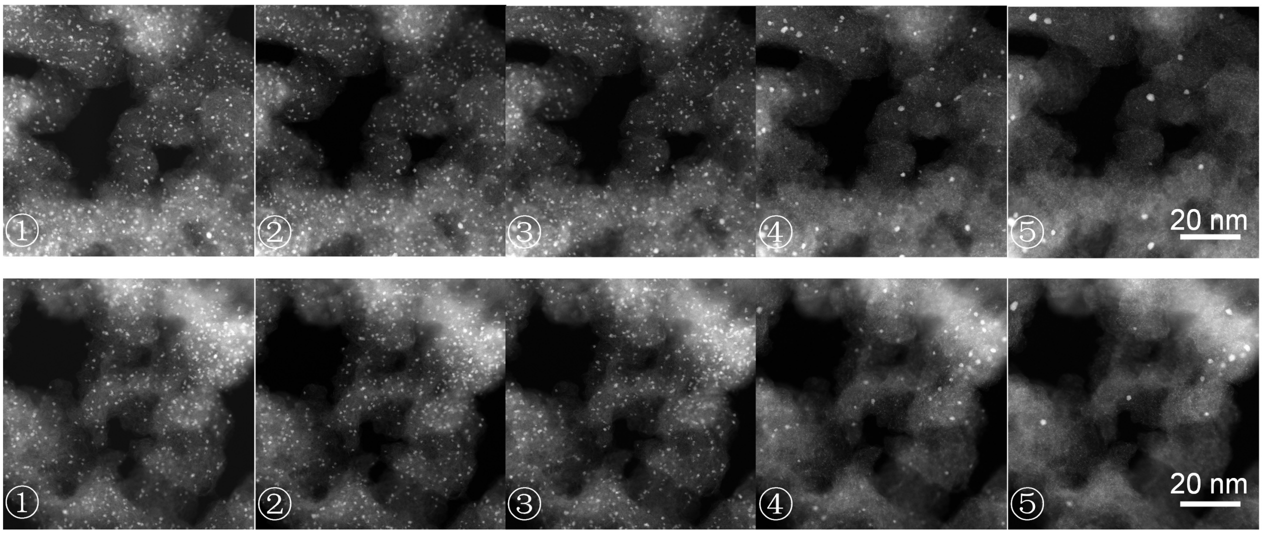

Supplementary Figure 17. In situ aberration-corrected HAADF-STEM images of 10%Pt/BP2000 ($d > d_c$) at five sintering stages shown in Fig. 4c.

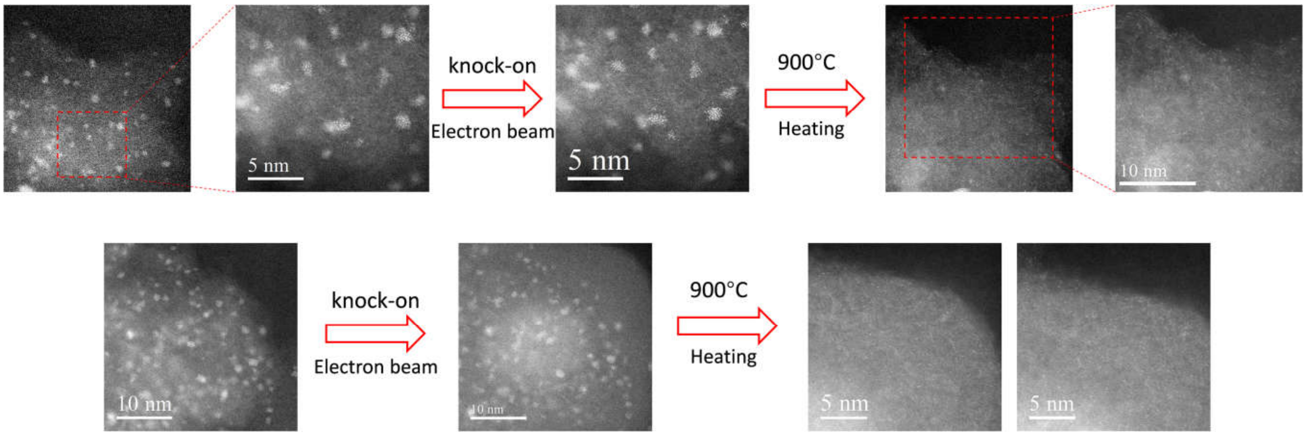

Supplementary Figure 18. Time-resolved STEM experiments with electron beam treatment for 30 min and then start heating at 900 °C.

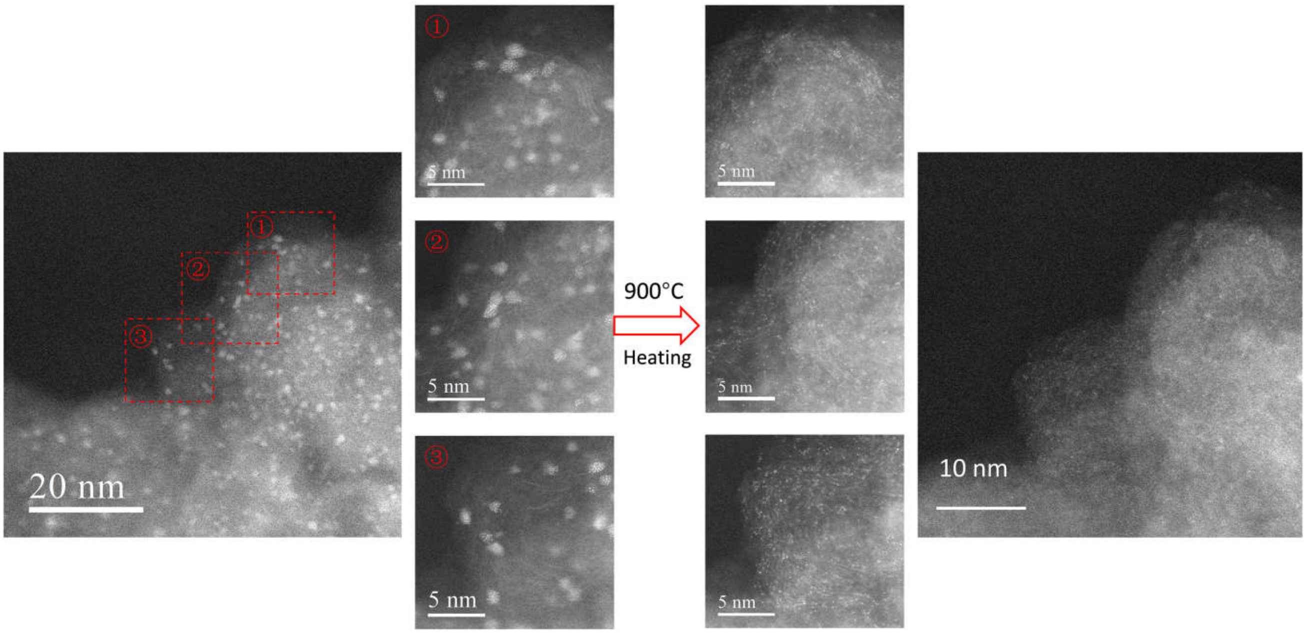

Supplementary Figure 19. In-situ heating STEM experiments, where the electron beam was closed during heating and only open to observe changes after the end of heating.

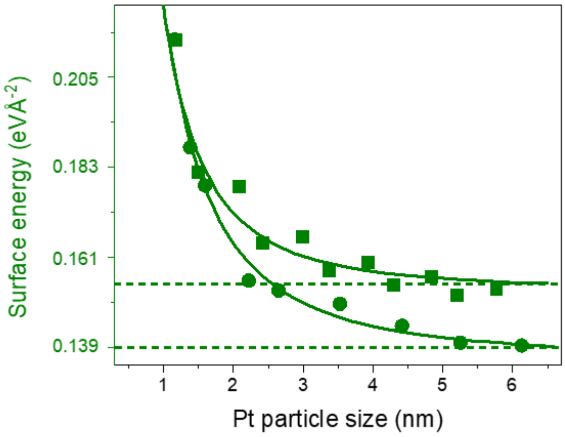

Supplementary Figure 20. Size dependence of surface energy of Pt nanoparticle based on constructed spherical (circles) and Wulff (squares) structures with a convergence to the bulk Pt surface energy (dashed line).

The surface energy per unit area $\gamma_{\mathrm{m}}(R)$ is size-dependent and could be calculated by Equation S1.

$$\gamma_{\mathrm{m}}(R) = \frac{\bar{C}^{\mathrm{m}}(R) - 12}{2 \times 12} \frac{E_{\mathrm{c}}}{S^{\mathrm{m}}} \qquad (1)$$

where $\bar{C}^{\mathrm{m}}(R)$ is the weighted surface coordination number for the Wulff structurem[1], S^{m} is the metal atom surface area and E_{c} is the cohesive energy of Pt. Accordingly, the size-dependent surface energy was confirmed and showed a convergence to the surface energy of bulk Pt (Supplementary Fig. S20).

The structure factor related with the contact angle α between the metal particle and support K_{α} could be calculated by Equation S2.

$$K_{\alpha} = \frac{3\nu_{\mathrm{p}} C_{\alpha} \Omega^2}{2\pi\alpha_1} \qquad (2)$$

$$C_{\alpha} = \begin{cases} \sin\alpha, & \alpha < \pi/2 \\ 1, & \alpha \geq \pi/2 \end{cases} \qquad (3)$$

where ν_{p} is the vibrational frequency of metal atom on support surface, R is the radius of curvature of nanoparticle on the support.

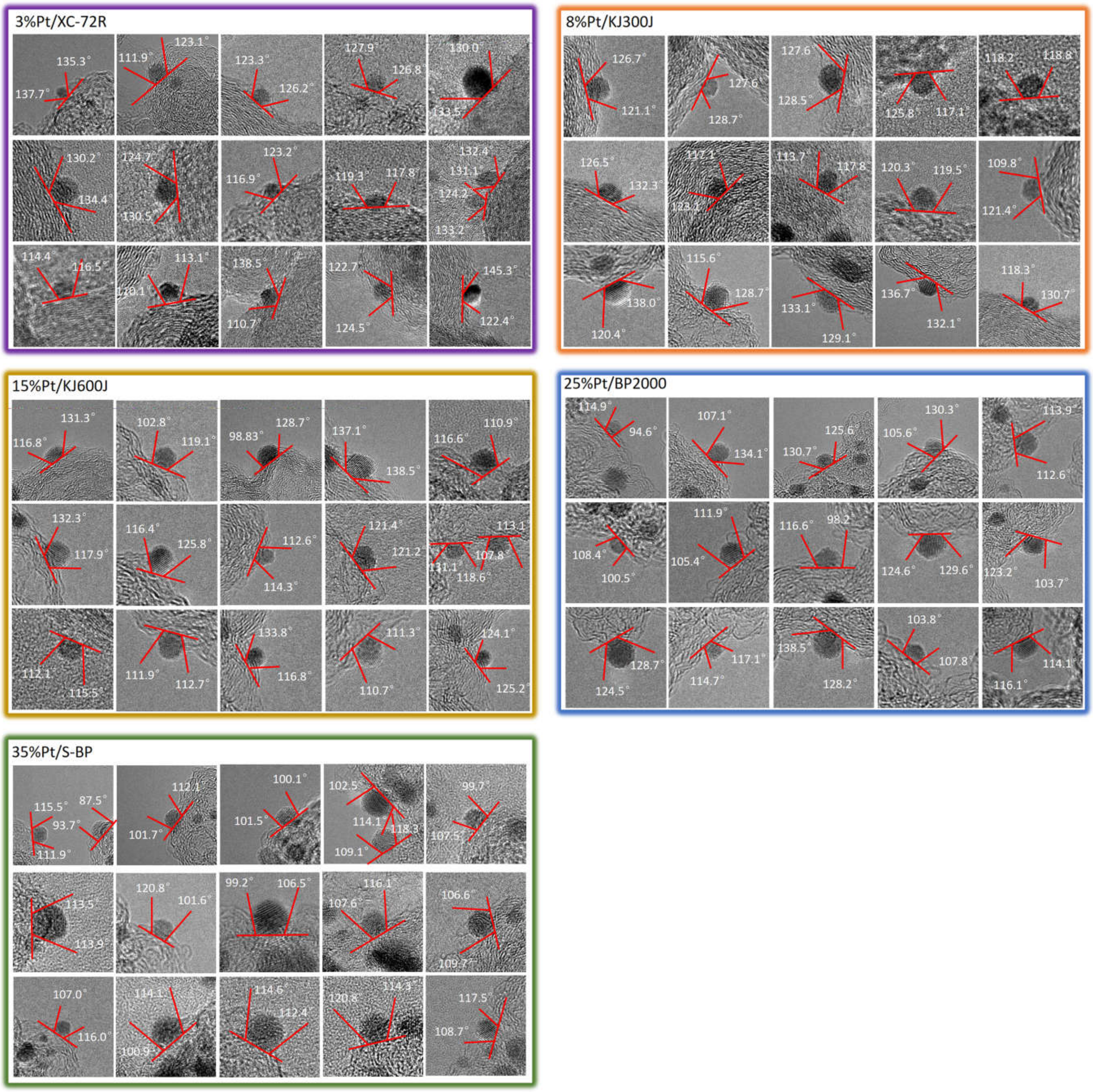

Supplementary Figure 21. Measured contact angle of Pt nanoparticles on the surface of the five carbon black supports.

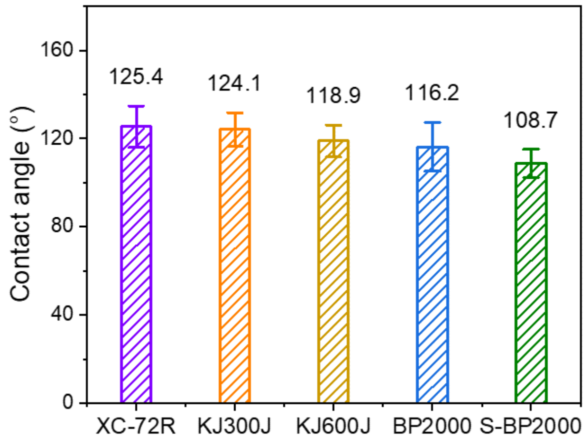

Supplementary Figure 22. Average contact angle of Pt nanoparticles on the surface of the five carbon black supports.

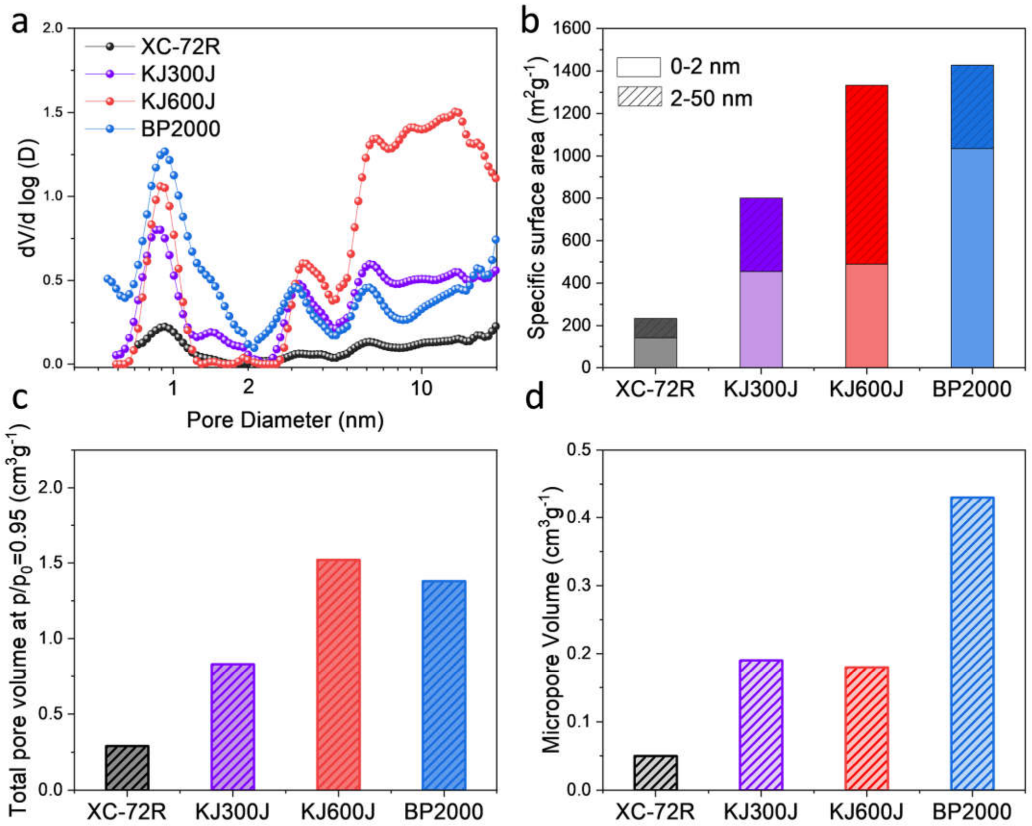

Supplementary Figure 23. Textural analyses of the four carbon black supports, including the pore size distribution (**a**), mesoporous specific surface areas (**b**), the total pore volume at p/p_0=0.95 (**c**), and the micropore volumes (**d**). XC-72R is a kind of solid carbons with little micropore and mesopore, while KJ300J, KJ600J, and BP2000 are hierarchically porous carbon with high specific surface areas. In particular, BP2000 has a much higher ratio of micropore volume and micropore surface area than KJ300J and KJ600J.

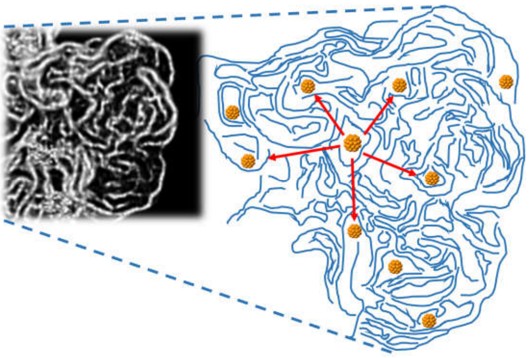

Supplementary Figure 24. Schematic illustration of complex microporous and mesoporous structure. Narrow channels of ~2 nm were observed connecting the internal carbon mesopores in carbon black supports, which means that Pt particles located in interior mesopores are not easy to agglomerate through the PMC mechanism owing to the obstruction of the narrow micropore channels at the outlet. Despite the absence of larger, mesopore-like openings, the Pt atoms of particles in the interior of the carbon supports may remain easily mobile through microporous channels via OR mechanisms. In this case, the abundance of micropores and complex pore structures increases the difficulty of mass transfer among particles via PMC/OR mechanisms.

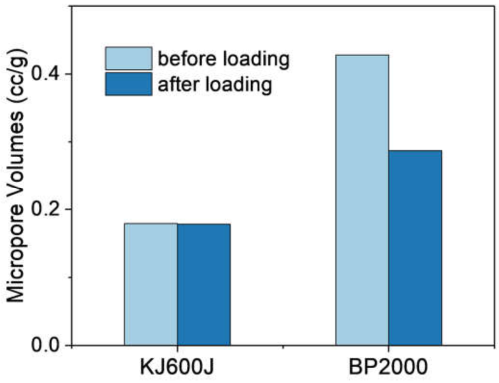

Supplementary Figure 25. Micropore volume changes before and after loading on KJ600J and BP2000. The micropore volume on Pt/KJ600 did not change much after loading, but the micropore volume of Pt/BP2000 obviously decreased. This result revealed that some micropores in BP2000 were occupied by Pt nanoparticles, which demonstrated some micropores were used to restrict small particles (< 2 nm) sintering on BP2000.

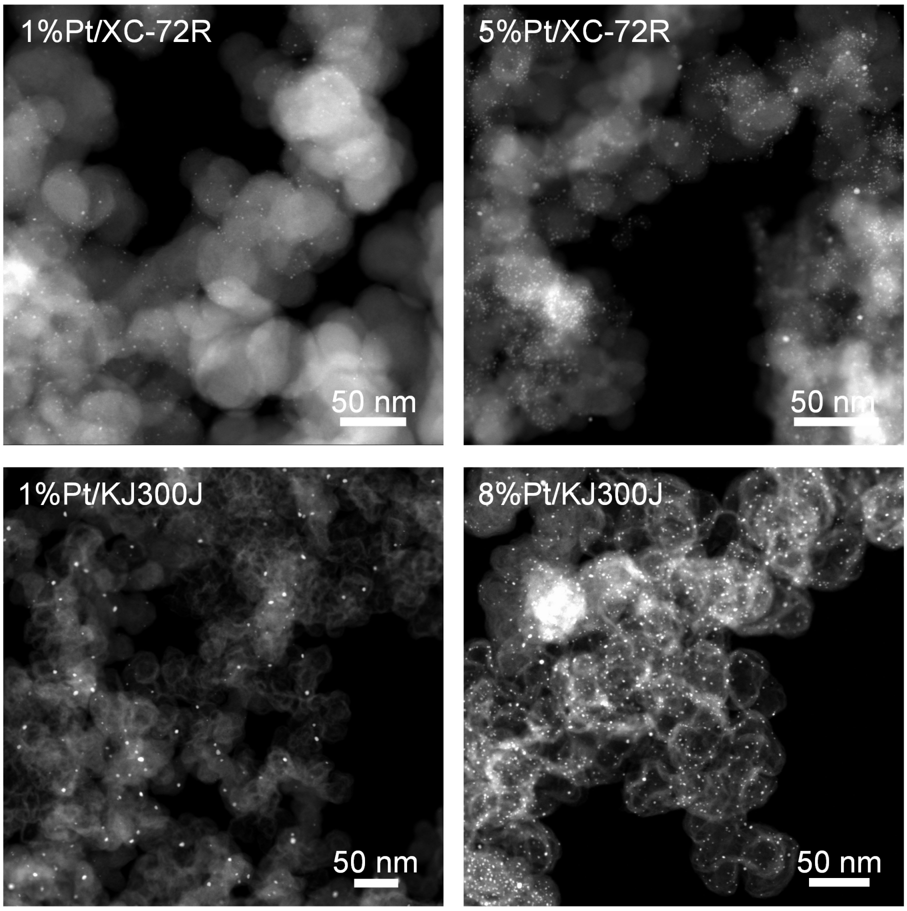

Supplementary Figure 26. HAADF-STEM images of the Pt/XC-72R (1%Pt and 5%Pt) and Pt/KJ300J (1%Pt and 8%Pt) catalysts.

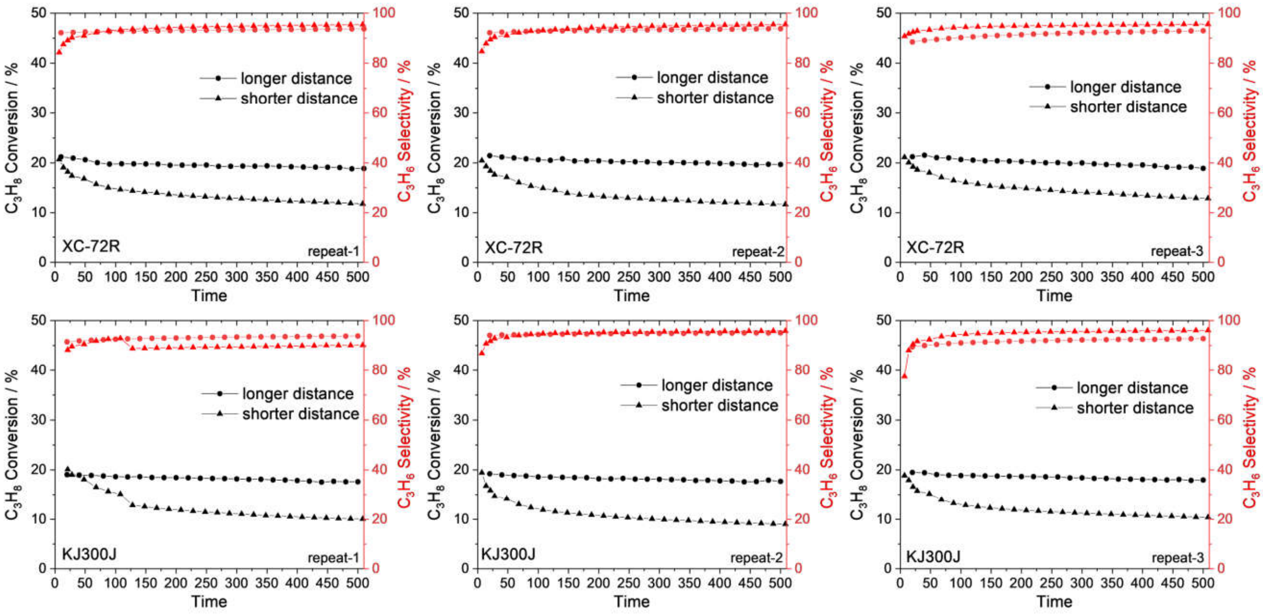

Supplementary Figure 27. Propane dehydrogenation performance of Pt/XC-72R and Pt/KJ300J at 500 ºC.

Supplementary Table 1. Instances of OR versus PMC of the Pt/C catalysts at early low-temperature and later high-temperature stages.

Sample	Stage	Number of particles	
		PMC	OR
10%Pt/XC-72R ($d < d_c$)	≤ 700 °C	96	35
	≥ 900 °C	13	37
1%Pt/XC-72R ($d > d_c$)	≤ 700 °C	0	60
	≥ 900 °C	0	2
10%Pt/XC-72R ($d > d_c$)	≤ 700 °C	0	65
	≥ 900 °C	0	5

Supplementary References

1 Dietze, E. M., Plessow, P. N. & Studt, F. Modeling the Size Dependency of the Stability of Metal Nanoparticles. *J. Phys. Chem. C* **123**, 25464-25469 (2019).
