# Supplementary material for: Quantification of critical particle distance for mitigating catalyst sintering
Source: Nat Commun. 2021 Aug 11;12:4865. doi: 10.1038/s41467-021-25116-2 (PMC8358017; doi:10.1038/s41467-021-25116-2)
Supplement: Supplementary file 3 — Description of Additional Supplementary Files [file 41467_2021_25116_MOESM3_ESM.pdf]

Description of Additional Supplementary Files

File Name: Supplementary Movie 1
Description: A movie of in-situ HAADF-STEM observation showing the sintering of Pt-NPs at long particle distance from 300 °C to 900 °C under vacuum

File Name: Supplementary Movie 2
Description: A movie of in-situ HAADF-STEM observation showing the sintering of Pt-NPs under only electron beam treatment without heating.

File Name: Supplementary Movie 3
Description: A movie of in-situ HAADF-STEM observation showing the sintering of Pt-NPs under only electron beam treatment without heating.
